# Construction of a novel prognostic-predicting model correlated to ovarian cancer

**DOI:** 10.1042/BSR20201261

**Published:** 2020-08-07

**Authors:** Weichun Tang, Jie Li, Xinxia Chang, Lizhou Jia, Qi Tang, Ying Wang, Yanli Zheng, Lizhou Sun, Zhenqing Feng

**Affiliations:** 1 National Health Commission Key Laboratory of Antibody Technique, Nanjing Medical University, Nanjing, People’s Republic of China; 2 Department of Pathology, Nanjing Medical University, Nanjing, People’s Republic of China; 3 Department of Nursing, The Second Affiliated Hospital of Nantong University, Nantong, People’s Republic of China; 4 Department of Gynaecology and Obstetrics, The Second Affiliated Hospital of Nantong University, Nantong, People’s Republic of China; 5 Department of Obstetrics and Gynecology, First Affiliated Hospital of Nanjing Medical University, Nanjing, People’s Republic of China

**Keywords:** bioinformatics, biological function, hub genes, Ovarian cancer, prognostic model

## Abstract

**Background**: Ovarian cancer (OC) is one of the most lethal gynecological cancers worldwide. The pathogenesis of the disease and outcomes prediction of OC patients remain largely unclear. The present study aimed to explore the key genes and biological pathways in ovarian carcinoma development, as well as construct a prognostic model to predict patients’ overall survival (OS).

**Results:** We identified 164 up-regulated and 80 down-regulated differentially expressed genes (DEGs) associated with OC. Gene Ontology (GO) term enrichment showed DEGs mainly correlated with spindle microtubes. For Kyoto Encyclopedia of Genes and Genomes (KEGG) pathways, cell cycle was mostly enriched for the DEGs. The protein–protein interaction (PPI) network yielded 238 nodes and 1284 edges. Top three modules and ten hub genes were further filtered and analyzed. Three candidiate drugs targeting for therapy were also selected. Thirteen OS-related genes were selected and an eight-mRNA model was present to stratify patients into high- and low-risk groups with significantly different survival.

**Conclusions:** The identified DEGs and biological pathways may provide new perspective on the pathogenesis and treatments of OC. The identified eight-mRNA signature has significant clinical implication for outcome prediction and tailored therapy guidance for OC patients.

## Background

Ovarian cancer (OC) is the most lethal malignant disease in the female reproductive system, with over 200000 new cases and 150000 deaths each year worldwide [1]. Epithelial OC accounts for 80–95% of ovarian malignancies, listed as the most common histological type. Since the ovaries locate in the deep pelvis, with mere symptoms emerging at the beginning of ovarian morbid change, the early detection for the malignancy is truly difficult. Hence, when OC is detected, the patient is usually at an advanced stage, with invasion and metastasis accompanied [2]. For patients in the early stage, the 5-year survival rate can reach 85–90%, whereas, for advanced-stage patients, the number is mere ∼20% [3]. Therefore, it is imperative to explore the molecular mechanisms of malignant biological behavior of OC cells and to develop more reliable molecular markers for predicting recurrence and evaluating prognosis, further guiding clinicians for therapy.

At present, various high-throughput microarrays and next-generation sequence genomic datasets, which were deposited in the Gene Expression Omnibus (GEO) [4] and The Cancer Genome Atlas (TCGA) databases, have been widely analyzed for identifying differentially expressed genes (DEGs), which could serve as candidate diagnostic or prognostic markers, further effectively improving our understanding of the disease from genetic perspective. Whereas, since the existence of tissue or sample heterogeneity in each independent experiment, as well as the discrepancy of different data processing methods and technology platforms, the DEGs identified from a single-cohort study may generate false positives. Herein, the Robust Rank Aggregation (RRA) method, which analyzes the significant probability of all elements by a probabilistic model, is developed to identify statistically significant genes from multiple datasets and provide more accurate and valuable information for clinical use far beyond one gene list [5]. To date, a bunch of novel prognostic markers have been discovered to potentially improve the efficacy of diagnosis and prognosis of OC [6]. However, these identified markers were only effective for partial stages or grades and were difficult to apply widely. Hence, a prognostic model, which is based on signature gene expression level, with high discriminating power to effectively assist prognosis prediction for each patient is required in clinical practice.

In the present study, we downloaded six primary microarray datasets from the GEO database which contained a total of 265 samples, with 201 OC samples and 64 normal samples. The gene-set and relative clinical information on ovary tissues of OC patients and healthy females from TCGA and GTEx portal were also downloaded. Integrated DEGs between cancerous and normal ovarian samples were screened using the ‘limma’ R package and RRA method. Gene Ontology (GO) and Kyoto Encyclopedia of Genes and Genomes (KEGG) pathways enrichment of DEGs were performed for next-step functional analysis. The Search Tool for the Retrieval of Interacting Genes Database (STRING) and the Connectivity Map (CMap) online database were then used to analyze the association of DEGs and explore the molecular mechanisms as well as drugs involved in tumorigenesis. Through survival analysis, prognostic mRNAs were also selected. By performing Cox regression analysis, we identified an eight-mRNA signature model with the ability to predict the prognosis of OC patients and independent from clinical factors. Our study provides reliable molecular markers and prognostic models for early detection and outcome prediction, as well as effective drug targets for treating OC.

## Methods

### Data collection

Through searching on the GEO Repository with ‘ovarian cancer’, we downloaded the gene expression profiles of GSE54388, GSE40595, GSE38666, GSE27651, GSE18520 and GSE14407 and the corresponding annotation files from the GPL570 [HG-U133_Plus_2] Affymetrix Human GenomeU133 Plus 2 Array platform. GSE54388 contains 22 ovarian tissue samples, with 6 normal ovarian surface epithelium and 16 tumor epithelial components from high-grade serous OC patients. GSE40595 includes 77 OC-associated stroma and epithelium samples, which consist of 31 cancer-associated stroma samples and 32 epithelial tissues from high-grade serous OC patients, along with 8 stromal component and 6 ovarian epitheliums from the normal ovary. GSE38666 comprises 8 stroma and 8 matched ovarian epitheliums from 12 healthy females, as well as 7 cancer stroma and 7 matched cancer epitheliums from OC patients. GSE27651 incorporates 6 normal ovarian surfaces epithelial and 8 serous borderline ovarian tumors, 13 low-grade serous ovarian carcinomas, and 22 high-grade serous ovarian carcinomas. GSE18520 covers 53 advanced stage, high-grade primary OC specimens and 10 normal ovarian surface epithelium tissues. GSE14407 involves 12 healthy ovarian surface epithelial samples and paired serous OC epithelium. Note that all samples from these GEO datasets are classified into the cancerous or normal part, to be clear, the normal stromal and surface epithelium is defined as normal ovarian tissues, whereas the borderline tumors as well as cancerous stromal and epithelial tissues are considered as malignancies. Besides, we also downloaded the FPKM format gene expression data and relative clinical information of 374 OC patients’ samples and normal ovarian tissues from TCGA and GTEx portal, respectively.

### Screening for DEGs and integration of microarray data

We used the ‘limma’ R package [7] to (1) integrate the expression profiles from TCGA and GTEx portal, (2) standardize the data from the integrated TCGA and GTEx expression matrix as well as six GEO datasets, and (3) further screen the DEGs between ovarian cancerous and normal samples. The list of DEGs obtained from six GEO microarray datasets by limma analysis was further integrated by the ‘RRA’ method to get prioritized commonly up- or down-regulated gene list. The final overlapped DEGs for subsequent biological function analysis were the combination of prioritized jointly dysregulated genes from six GEO microarrays and the results from TCGA and GTEx databases. The cut-off criteria were set as FDR < 0.05 and |log2fold change (FC)| > 1.

### GO term and KEGG pathway enrichment analysis

GO classified the known genes into three main biological progress: Molecular Function (MF), Cellular Component (CC) and Biological Process (BP) [8]. KEGG provides researchers high-layer functions of the biological system from molecular level information [9]. The Enrichr online tool (https://amp.pharm.mssm.edu/Enrichr/) allows for GO term annotation and KEGG pathway for a cluster of genes [10,11]. We explored the biological functions of overlapped DEGs and hub modules from our protein–protein interaction (PPI) network using Enrichr website. *P*-value <0.05 was considered as significant enrichment. Likewise, the functional biological pathways of the top ten hub genes from PPI network were also analyzed by the FunRich tool (version: 3.0) [12] and the top five enriched pathways of up- and down-regulated genes were displayed as bar charts, respectively. We set the *P*-value <0.05 as statistically significant.

### PPI network construction and analysis

PPI networks display the relationships of various proteins according to their physical or biochemical properties. STRING is a database that encompasses the interaction information between known proteins and potentially interacted proteins [13]. In order to explore the correlations between the DEGs, we used the STRING database to construct a PPI network and visualize our results by Cytoscape software [14]. Confidence score > 0.4 was set as significant. Molecular Complex Detection (MCODE) was utilized to select hub modules of PPI networks in Cytoscape [15]. We set the degree cut-off = 2, node score cut-off = 0.2, k-score = 2 and max. Depth = 100 was set as the criterion. Then, the significant modules were performed by GO and KEGG analyses. Top ten genes were defined according to the high degree of connectivity in STRING network. [16]. The co-expression analysis of ten hub genes was performed by STRING, either.

### Validation of the hub genes

We downloaded the raw gene-set of OC patients from TCGA to explore the expression differences of hub genes in low- and high-grade tumor tissues of OC and draw the survival plot using Kaplan–Meier plotter webtool [17]. The gene and protein expression level of grade-related hub genes were then confirmed by Oncomine and The Human Protein Atlas (HPA) database [18,19]. Meanwhile, we explored the genetic alteration information of the selected ten hub genes in OC patients by plug-in cBioPortal (cBio Cancer Genomics Portal) tool, which deposits the genomics profiles of various cancer types and provides analysis and visualization of the genomics datasets [20].

### Identification of candidate small molecule drugs

The CMap database was able to predict potential drugs which might reverse, or induce the biological state encoded in certain gene expression signatures in OC [21]. The 244 DEGs from our study were used to query the CMap database. The enrichment scores which represent the similarity were calculated (ranging from −1 to 1). The positive connectivity score means an inducing influence on the input signature, whereas drugs with negative connectivity score present reversion impact on the characteristic in human cell lines and are considered as candidate therapeutic molecules. After sorting all instances, the connectivity score of various instances was filtered by *P*-value <0.05. Next, we investigate the structures of these candidate molecular drugs from the Pubchem database (https://pubchem.ncbi.nlm.nih.gov/).

### Establishment and evaluation of the prognostic model

The 374 OC patients from the TCGA project were randomly classified into the training cohort (*n*=188) and the testing cohort (*n*=186). OS-related genes were determined by performing univariate Cox regression analysis in the training cohort with the ‘Survival’ R package and further selected for the next-step screening. Least Absolute Shrinkage and Selection Operator (LASSO) is a parameter selection algorithm which shrinks all high-dimensional regression coefficients and generates the penalty regularization parameter λ via the cross-validation routine by ‘glmnet’ R package. To select the optimal prognostic mRNAs, we adopted LASSO regression among the selected candidate genes and further perform multivariate Cox proportional hazards regression to evaluate their independent prognostic values. The risk-score model for predicting outcomes of OC patients was the sum of each optimal prognostic mRNA expression level multiplying relative regression coefficient weight calculated from the multivariate Cox regression model.

$$\text{Risk score }(\text{patient})\;=\;\underset{i}{\sum \;}{\text{Coefficient (mRNA}}_{i}\text{) }\times \;\text{Expression }\left({\text{mRNA}}_{i}\right)$$

All training cohort patients were classified into high- and low-risk groups according to the median risk score. The Kaplan–Meier curves of two diverse groups were plotted using ‘survfit’ function and the receiver operating characteristic (ROC) curve was unfolded for OS prediction to estimate the sensitivity and specificity of the prognostic model. Cox multivariate analysis was also performed to examine whether the risk score was independent of the clinical characters, such as age, tumor stage and grade. Next, we used the testing group to check the efficacy of the prognostic risk model. Each individual in the testing cohort was also categorized as high- or low-risk case by comparing the patient’s risk score with the cut-off value calculated from the training cohort. Kaplan–Meier curve analysis, time-dependent ROC analysis and Cox multivariate analysis were performed, either.

### Searching tumor-infiltrating immune cells associated with patients' prognostic signatures

The TIMER webtool allows for systematical evaluations of the relationship between the six immune cell types in the tumor microenvironment which are B cell, CD4 T cell, CD8 T cell, neutrophil, macrophage as well as dendritic cell, and clinical impact in various cancer types via a novel statistical method [22]. To further explore the prognostic signature, we used the TIMER online tool to search the most significant tumor-infiltrating immune cells according to the TCGA OC gene data. To be clear, the relative gene expression levels of six types of immune cells for each patient in high- and low-risk groups from training and testing cohort were measured.

## Results

### The DEGs among six GEO microarray datasets

**Figure 1:**
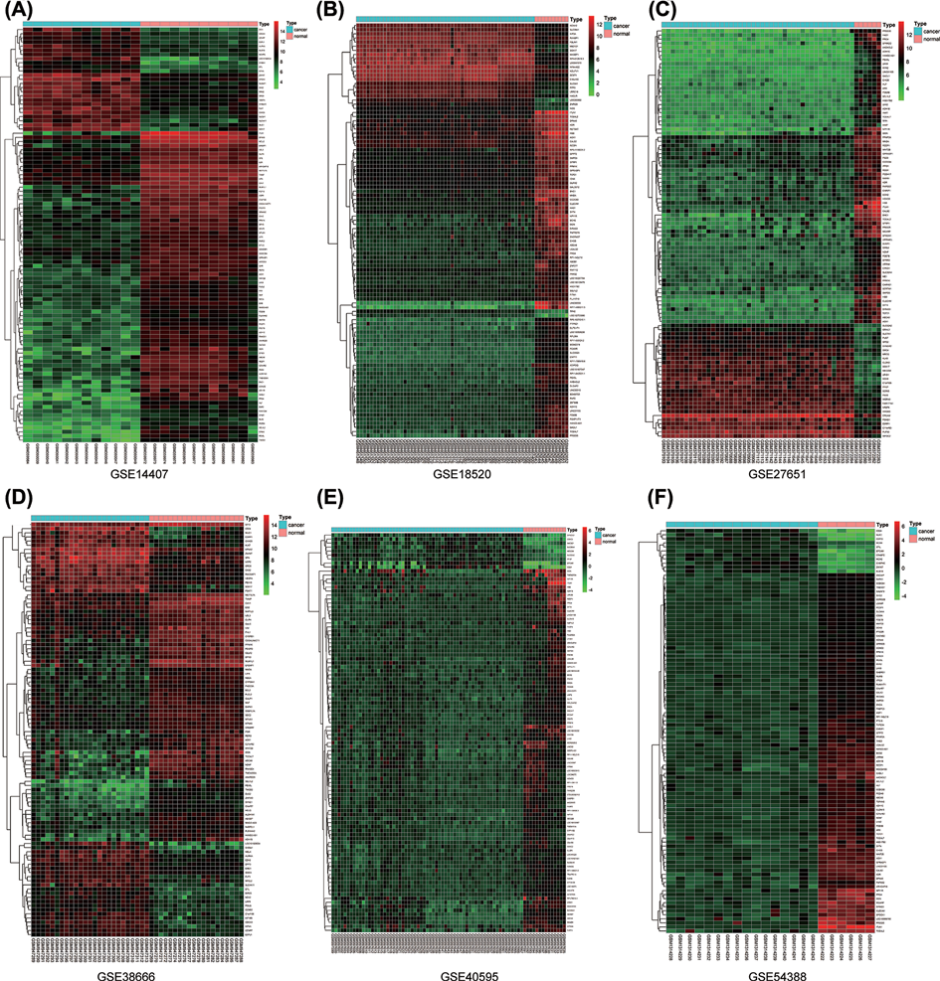
The expression heatmap of the top 100 significantly dysregulated genes in six GEO datasets Hierarchical clustering that shows the expression profiles of mRNAs from (**A**) GSE14407, (**B**) GSE18520, (**C**) GSE27651, (**D**) GSE38666, (**E**) GSE40595, (**F**) GSE54388.

**Figure 2:**
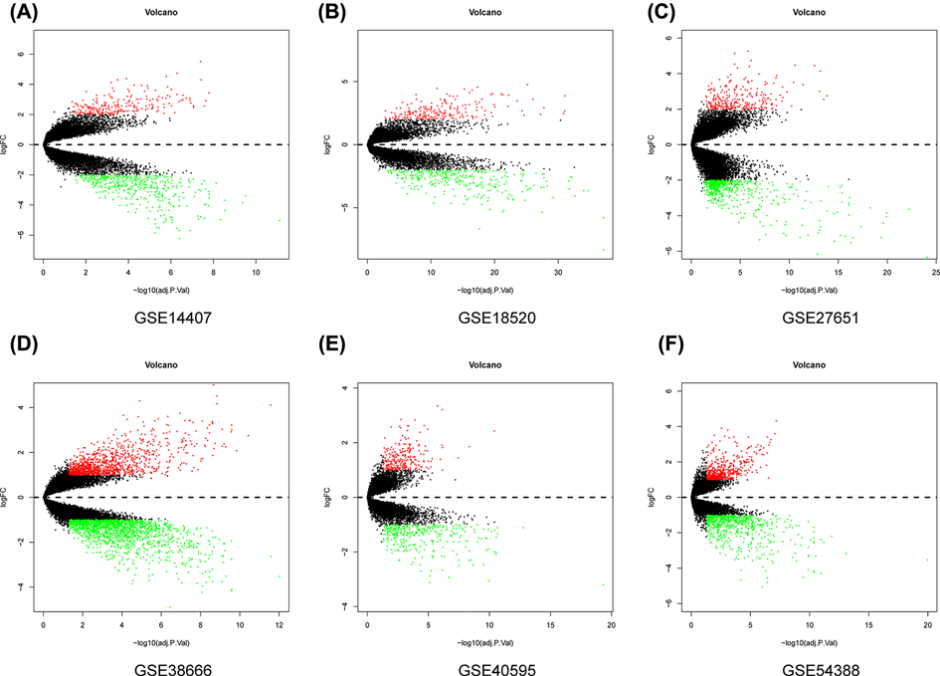
The volcano plot of all gene expression distribution in six GEO datasets Volcano plot of differentially expressed mRNAs of (**A**) GSE14407, (**B**) GSE18520, (**C**) GSE27651, (**D**) GSE38666, (**E**) GSE40595, (**F**) GSE54388.

**Figure 3:**
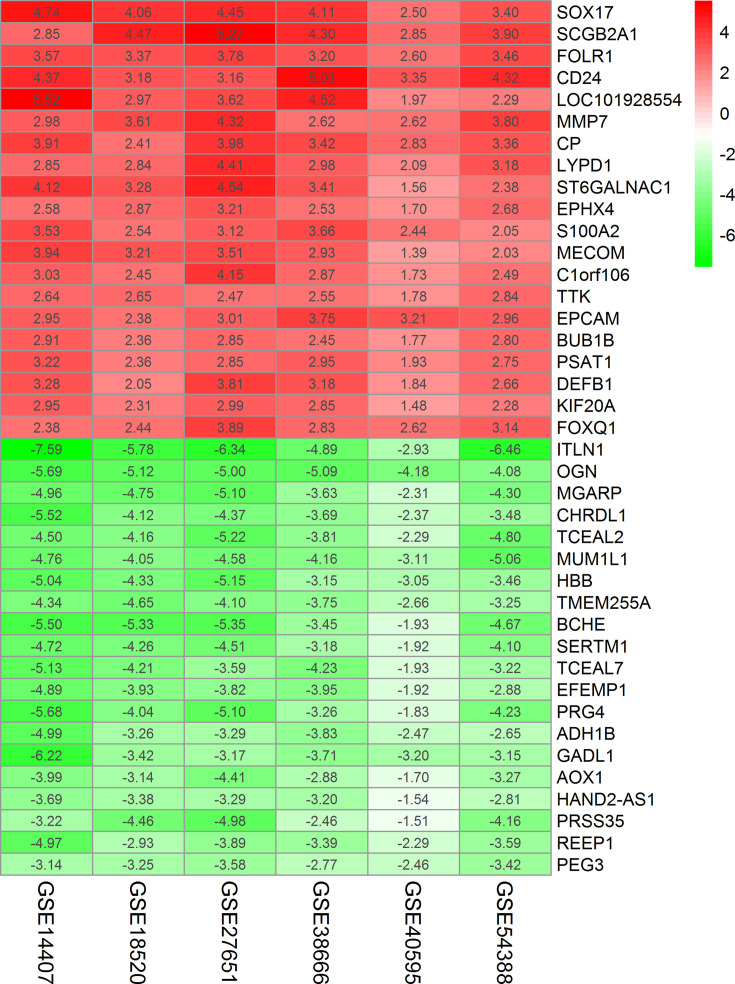
Heatmap showing the top 20 up-regulated genes and top 20 down-regulated genes according to *P*-value Each row represents one gene and each column indicates one dataset. Red indicates up-regulation and blue represents down-regulation. The numbers in the heatmap indicate log |FC| in each dataset calculated by the ‘limma’ R package. Abbreviation: log |FC|, logarithmic fold change.

**Figure 4:**
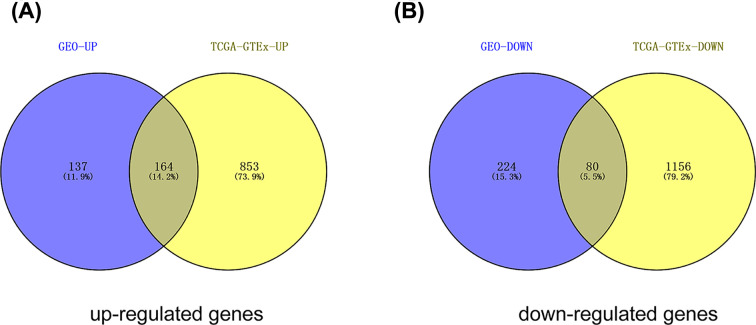
The intersection of up- and down-regulated DEGs of GEO and TCGA datasets (**A**) upregulated intersected DEGs in both datasets; (**B**) down-regulated intersected DEGs in both GEO and TCGA dataset. The intersected DEGs were defined as the significant DEGs.

The top 100 significantly up- and down-regulated genes from each microarray dataset were displayed in the heatmaps (Figure 1A–F) and the distribution of all gene expression was presented in volcano plots (Figure 2A–F). Through RRA analysis of 6 expression microarrays, we identified 605 DEGs, which consisted of 301 up-regulated and 304 down-regulated genes, and displayed the top 20 dysregulated genes by ‘pheatmap’ R package in Figure 3. Next, we analyzed the expression profiles of TCGA and GTEx, getting 2253 dysregulated genes. Intriguingly, when these 2253 DEGs were combined with the 605 DEGs from GEO datasets, we found that 244 genes were commonly dysregulated in these two databases, with 164 up-regulated (Figure 4A) and 80 down-regulated genes (Figure 4B).

### GO term and KEGG pathway enrichment analysis of DEGs

**Figure 5:**
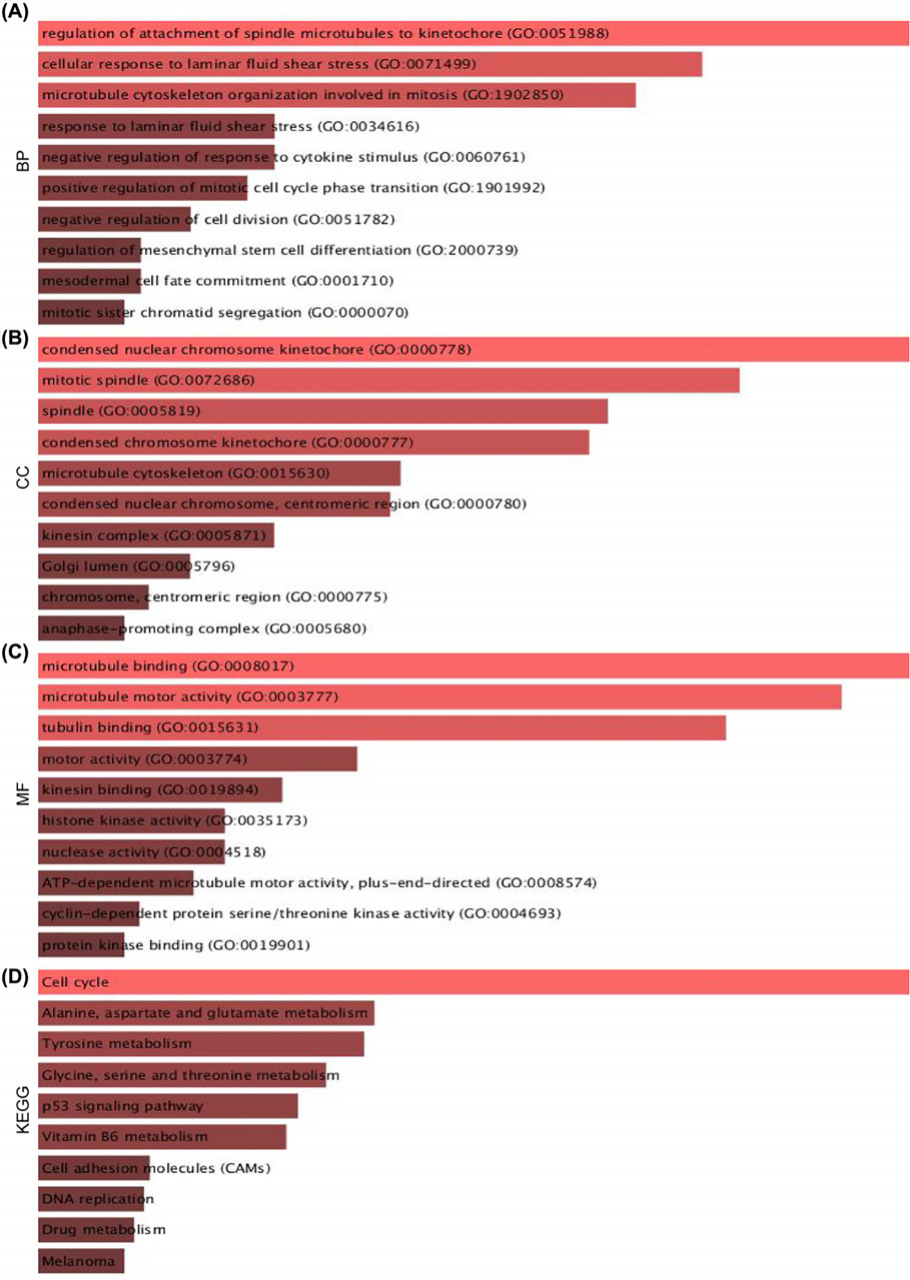
GO and KEGG functional annotation for the significant DEGs (**A**) The top ten enriched BP of the DEGs. (**B**) The top ten enriched CC of the DEGs. (**C**) The top ten enriched MF of the DEGs. (**D**) The top ten enriched KEGG pathways of the DEGs.

To study the potential biological function of the 244 DEGs, we performed biological pathway analysis and identified significantly enriched pathways via Enrichr web tools. In GO term (Figure 5A), for the BP group, the DEGs were mostly enriched in ‘regulation of attachment of spindle microtubes to kinetochore’, ‘cellular response to laminar fluid shear stress’ and ‘microtubule cytoskeleton organization involved in mitosis’. In MF group, the dysregulated genes were highly correlated to ‘microtubule-binding’, ‘microtubule motor activity’ and ‘tubulin-binding’. As for CC group, the DEGs were closely related to ‘condensed nuclear chromosome kinetochore’ and ‘mitotic spindle’. KEGG pathway analysis showed 244 DEGs highly enriched in ‘cell cycle’ and ‘Alanine, aspartate, and glutamate metabolism’ (Figure 5B).

### PPI network construction and modules analysis

**Figure 6:**
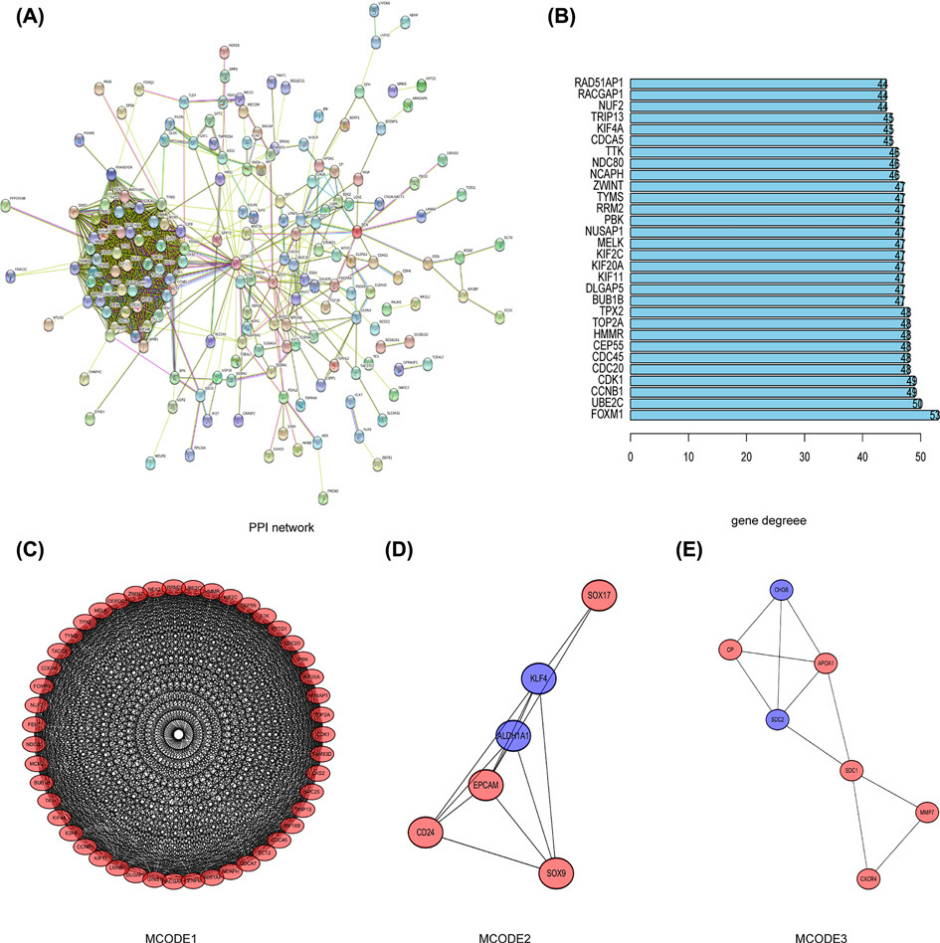
The PPI network and top 30 hub genes, as well as top three modules were constructed (**A**) The PPI network of the DEGs. (**B**) The 30 hub genes of the DEGs. (**C–E**) Top three hub modules were identified by Cytoscape plug-in MCODE: (C) module-1; (D) module-2; (E) module-3.

Using the STRING database and Cytoscape software, a total of 244 DEGs were mapped into the PPI network, which included 238 nodes and 1284 edges (Figure 6A). The PPI enrichment *P*-value was <1.0e-16. The top three key modules (Figure 6C–E) within PPI network were then selected (Module 1, MCODE score = 43.391; Module 2, MCODE score = 4.8; Module 3, MCODE score = 3.667) and the biological function of Module 1, which consisted of 47 nodes and 998 edges, was further analyzed. GO analysis indicated that Module1 was mainly associated with ‘regulation of attachment of spindle microtubules to kinetochore’, ‘condensed nuclear chromosome kinetochore’ and ‘microtubule motor activity’. KEGG analysis showed that ‘cell cycle’ and ‘oocyte meiosis’ were the most highly enriched pathways (Supplementary Figure S1).

### The screening of Hub genes and their characteristics

**Figure 7:**
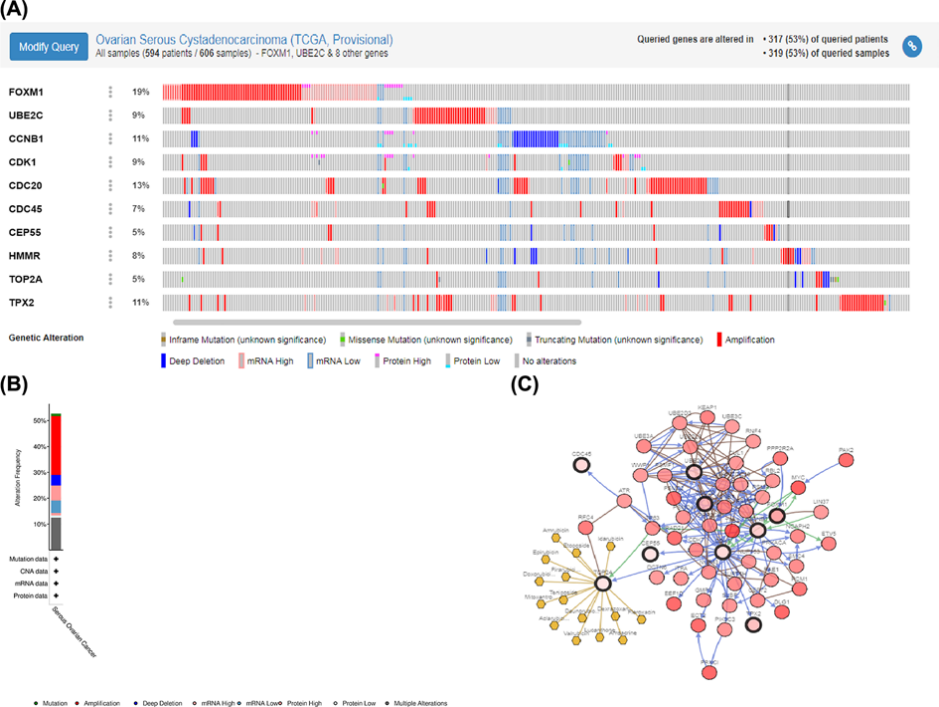
The gene mutation overview of ten hub genes in TCGA OC patients (**A**) Ten hub genes are altered in 317 (53%) of queried patients. (**B**) The summary of mutation type of ten hub genes in OC patients. (**C**) The network of 10 hub genes and the 50 most frequently altered neighbor genes.

**Figure 8:**
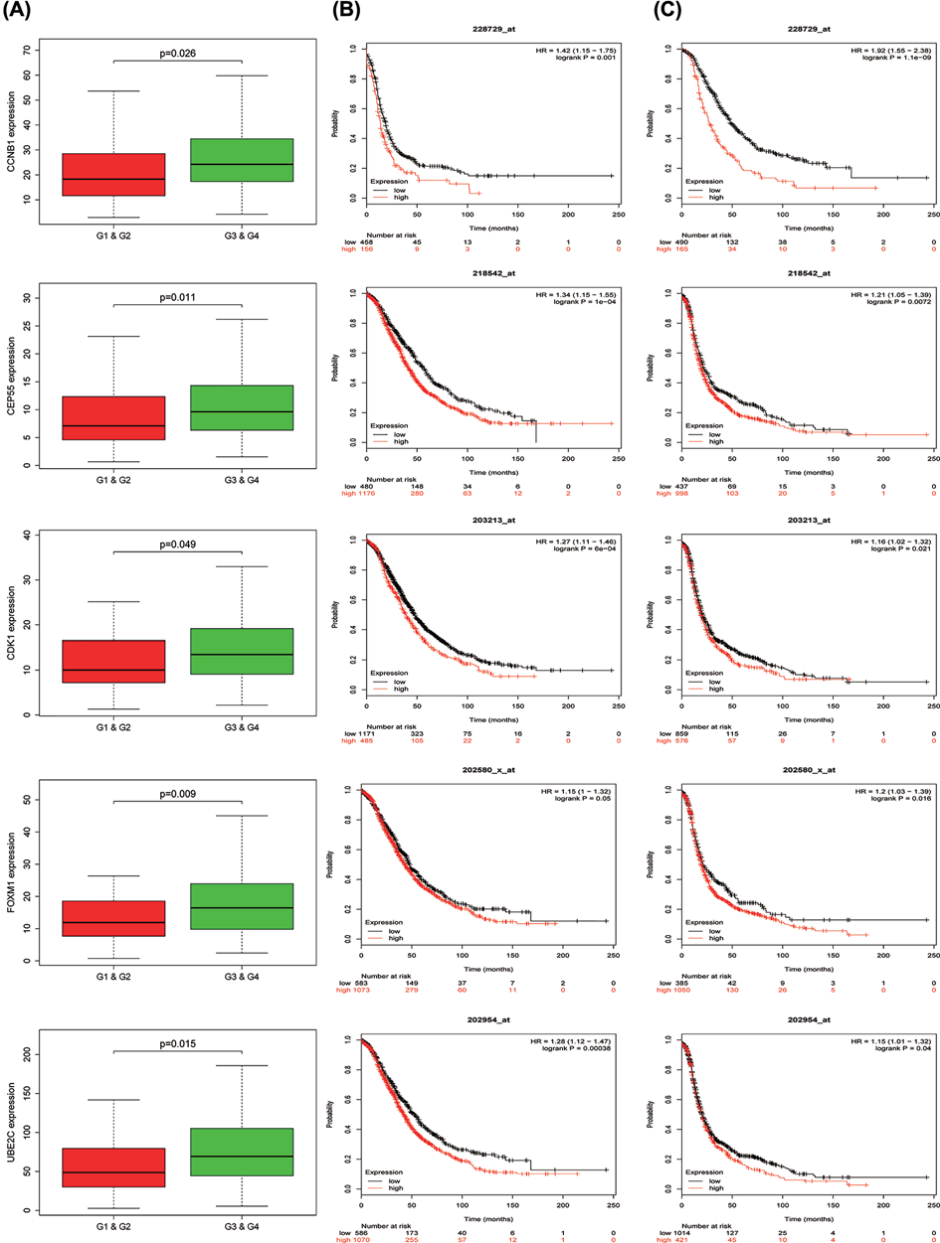
The clinical characteristics of CCNB1, CEP55, CDK1, FOXM1 and UBE2C in OC patients (**A**) Five genes were overexpressed in high grade (G1 and G2) compared with low grade (G3 and G4) in OC. (**B,C**) The OS time (**B**) and DFS time (**C**) of five genes in OC patients.

The top ten hub genes with the highest degree of connectivity were *CDC45, CDK1, TOP2A, CDC20, CCNB1, CEP55, UBE2C, HMMR, FOXM1* and *TPX2* (Figure 6B). The co-expression analysis results of the hub genes demonstrated that these genes actively interacted with each other (Supplementary Figure S2). Besides, we established the interaction network of ten hub genes with their related genes and explored the biological role (Supplementary Figure S2A,C–F) of the network by FunRich. The gene alteration type and frequency, as well as the 50 most frequently altered neighbor genes were also exhibited (Figure 7). Gene alteration frequency of ten hub genes among 606 TCGA OC samples was beyond 50%, with most genes showed amplified and multiple altered (Figure 7A,B). The top three most frequently altered genes were FOXM1, CDC20 and CCNB1, with FOXM1 and CDC20 largely amplified, while CCNB1 deep deleted. Through analysis of OC patients’ gene-set from TCGA, we found that CCNB1, UBE2C, CDK1, CEP55 as well as FOXM1 expressed significantly higher in high-grade tumors and predicted worse outcomes since patients overexpressed above genes owned lower overall survival (OS) and disease-free survival (DFS) rates (Figure 8). The Oncomine database showed results from various studies were consistent to our finding (Supplementary Figure S3). The HPA website also demonstrate that proteins translated by such five hub genes were overexpressed in OC tissues (Supplementary Figure S4). HMMR and TPX2 were also negatively correlated to patients’ prognosis while no expression difference was observed in diverse tumor grades and CDC20 was positively associated with tumor grade but not correlated to patients’ outcomes.

### Related small molecule drugs screening

In total, 244 DEGs were analyzed in CMap to screen small molecule drugs, and the candidate molecules with top ten connectivity score are listed in Table 1. Five of these molecules showed a negative correlation and suggested potential in clinical applications. Among them, Trichostatin A, pyrvinium and 8-azaguanine showed a significantly negative correlation and the stuctures of such candidate molecule drugs was found in the PubChem database and shown in Supplementary Figure S5.

**Table 1:** The top ten OC-related small molecules with highly significant correlations in results of CMap analysis

Rank	CMap name	Mean	N	Enrichment	P value
1	trichostatin A	−0.468	182	−0.448	0
2	8-azaguanine	−0.749	4	−0.913	0.0001
3	pyrvinium	−0.619	6	−0.772	0.00026
4	isoflupredone	0.7	3	0.937	0.00042
5	quinpirole	0.628	4	0.866	0.00044
6	vorinostat	−0.539	12	−0.558	0.00054
7	genistein	0.3	17	0.47	0.00064
8	antimycin A	−0.632	5	−0.788	0.00082
9	heptaminol	0.586	5	0.797	0.00084
10	midodrine	0.547	5	0.792	0.00086

### Construction of prognostic model and evaluation of its predictive ability

**Figure 9:**
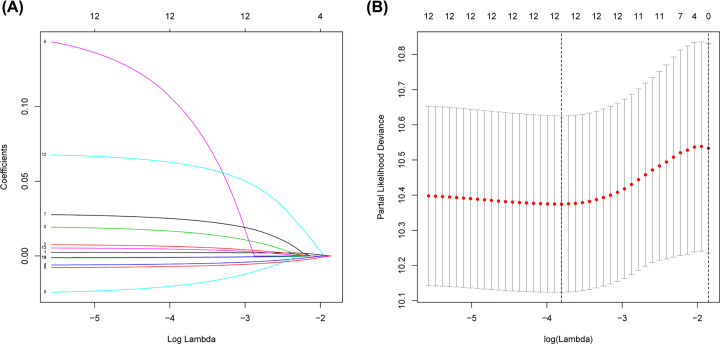
Identification of prognosis-related mRNAs using LASSO regression model (**A**) LASSO coefficient profiles of the mRNAs associated with the OS of OC. (**B**) Plots of the cross-validation error rates. Each dot represents a λ value along with error bars to give a confidence interval for the cross-validated error rate.

**Figure 10:**
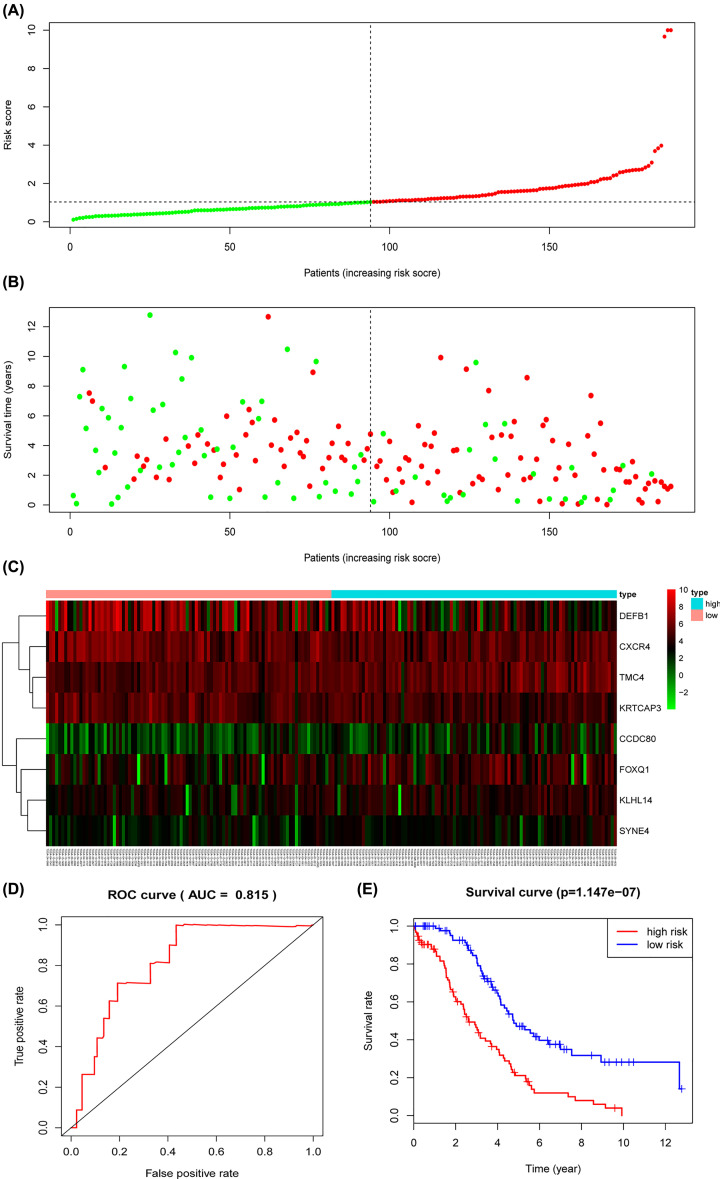
Prognostic analysis of the TCGA training model (**A**) The risk score, (**B**) survival status, (**C**) expression heatmap, (**D**) time-dependent ROC curves and (**E**) Kaplan–Meier survival of the prognostic model for the TCGA OC training cohort.

**Figure 11:**
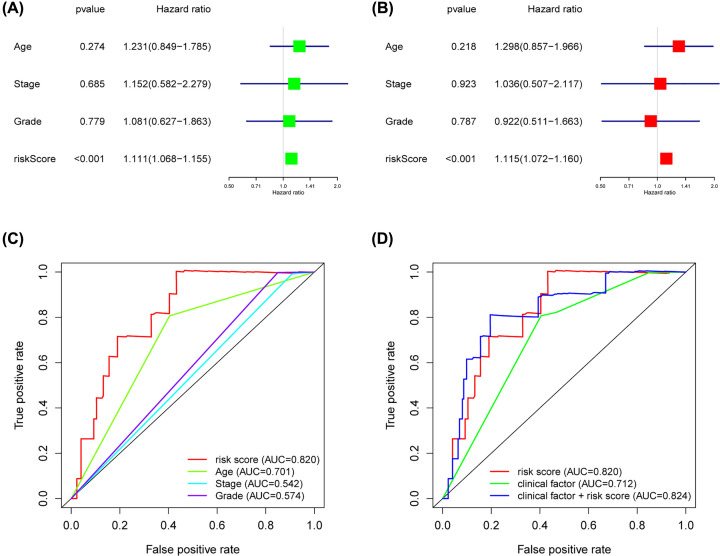
The clinical independency of the risk model in training cohort Univariate (**A**) and multivariate (**B**) regression analyses, as well as time-dependent ROC curve analysis (**C,D**) of the prognostic value between the training model and OC patients’ OS status when compared with or combined with clinical factors.

Univariate Cox regression analysis revealed that 13 of 244 DEGs were significantly correlated to patients’ OS in the training cohort (Table 2). The 13 OS-related genes were listed as follows: *CCND1, SYNE4, CCDC80, TMC4, MCC, FOXQ1, KRTCAP3, CXCR4, IL4I1, DEFB1, CSGALNACT1, KLHL14* and *MCUR1*. Through LASSO Cox regression, we narrowed the number of 13 prognosis-associated genes to 12 according to the minimum criteria (Figure 9). Next, based on the multivariate Cox model, 8 of 12 candidate mRNAs retained their prognostic significance and were finally selected as independent remarkable prognostic factors, which were TMC4, KLHL14, CXCR4, CCDC80, KRTCAP3, DEFB1, SYNE4 and FOXQ1 (Table 3). To predict patients’ outcomes, we developed an individual’s risk score model as follows: risk score = (0.006809 × expression value of TMC4) + (0.021258 × expression value of KLHL14) + (−0.00839 × expression value of CXCR4) + (0.031459 × expression value of CCDC80) + (−0.00903 × expression value of KRTCAP3) + (−0.00156 × expression value of DEFB1) + (0.070689 × expression value of SYNE4) + (0.006726 × expression value of FOXQ1). On the basis of the median risk score, patients were divided into high- or low-risk groups. Kaplan–Meier curve analysis showed that the OS time of the low-risk group was significantly longer than the high-risk group (*P*=1.147e-07) (Figure 10E). ROC curve analysis revealed the area under the ROC curve (AUC) of the prognostic model was 0.815 (Figure 10D). Meanwhile, the risk scores (Figure 10A) of OC patients in the training group were ranked for displaying their distribution and the survival status (Figure 10B) was marked on the dot plot. The expression pattern of eight prognostic mRNAs between high and low-risk groups was also shown in the heatmap (Figure 10C). Univariate and multivariate Cox regression analyses concerning the risk score and clinical factors showed that the prognostic model was able to serve as an independent prognostic indicator (Figure 11A,B). ROC curve analysis also showed that the AUC value of the model was 0.820, much significantly higher than the tumor stage (AUC = 0.542), grade (AUC = 0.574) and patients’ age (AUC = 0.701) (Figure 11C). Interestingly, when combined the risk score with clinical factors, the ROC curve of combination model was much higher than each alone (Figure 11D).

**Table 2:** Univariate cox regression identified 13 DEGs correlated to patients’ OS

Gene ID	HR	HR.95L	HR.95H	*P*-value
*CCND1*	1.006326448	1.002566689	1.010100307	0.000959275
*SYNE4*	1.049235696	1.019307082	1.080043064	0.001133431
*CCDC80*	1.027423125	1.008550366	1.046649046	0.004235937
*TMC4*	1.008946557	1.002423207	1.015512357	0.007117928
*MCC*	1.158403166	1.035973925	1.29530084	0.0098773
*FOXQ1*	1.009835231	1.001811636	1.017923088	0.016186926
*KRTCAP3*	0.990364725	0.982252065	0.998544389	0.021051161
*CXCR4*	0.993964188	0.988729288	0.999226806	0.024636469
*IL4I1*	0.994202937	0.989120462	0.999311528	0.026193083
*DEFB1*	0.998104524	0.996327955	0.999884261	0.036860792
*CSGALNACT1*	1.430557852	1.014289405	2.017664543	0.041267552
*KLHL14*	1.020200309	1.000551278	1.040235213	0.043851588
*MCUR1*	0.953716572	0.910205203	0.999307955	0.046698901

Abbreviation: HR, hazard ratio.

**Table 3:** Multivariate Cox regression selected eight DEGs correlated to patients’ OS

Gene ID	HR	HR.95L	HR.95H	*P*-value
*TMC4*	1.006831852	0.999904624	1.01380707	0.053250159
*KLHL14*	1.021485339	1.001857759	1.041497446	0.031756629
*CXCR4*	0.991645394	0.985647879	0.997679404	0.006716471
*CCDC80*	1.031959471	1.011590262	1.052738832	0.001982146
*KRTCAP3*	0.991005724	0.982638972	0.999443715	0.03674419
*DEFB1*	0.998439683	0.996606595	1.000276142	0.095816854
*SYNE4*	1.073246946	1.045846994	1.101364745	8.45E-08
*FOXQ1*	1.006748837	0.998099969	1.015472649	0.126528285

Abbreviation: HR, hazard ratio.

**Figure 12:**
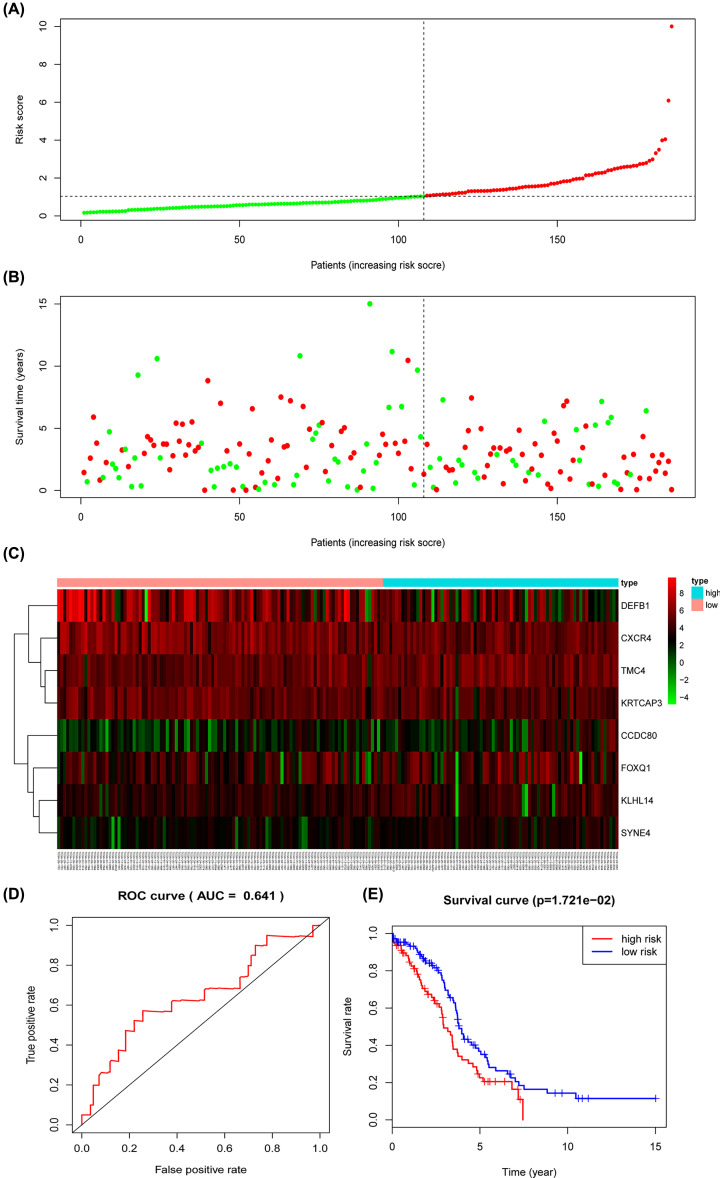
Prognostic analysis of the TCGA testing model (**A**) The risk score, (**B**) survival status, (**C**) expression heatmap, (**D**) time-dependent ROC curves and (**E**) Kaplan–Meier survival of the prognostic model for the TCGA OC testing cohort.

**Figure 13:**
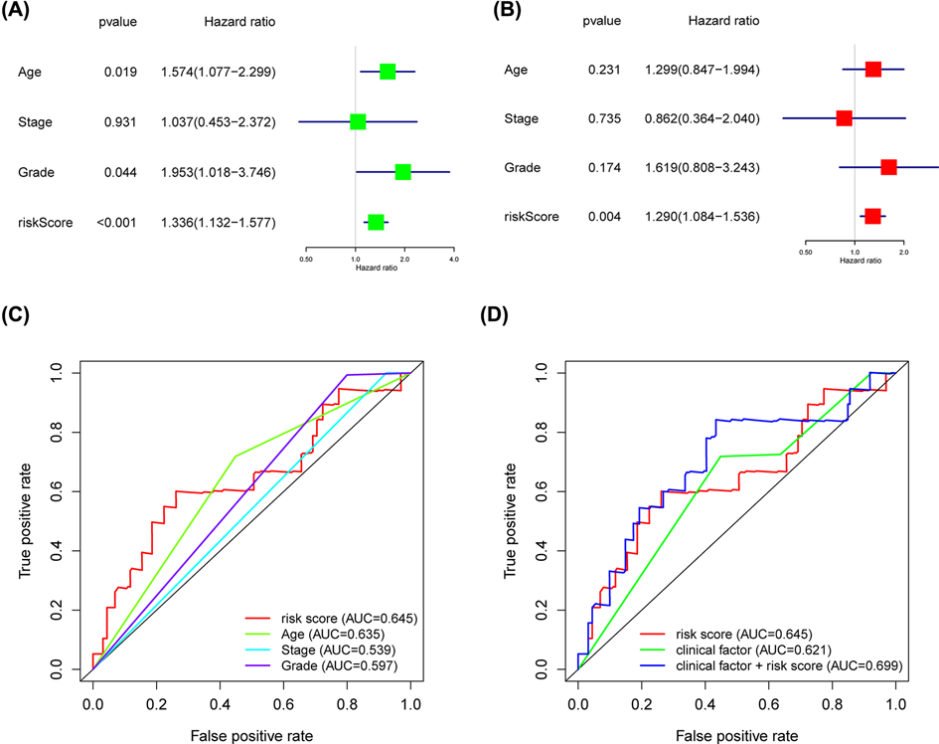
The clinical independency of the prognostic risk signature in testing cohort Univariate (**A**) and multivariate (**B**) regression analyses, as well as time-dependent ROC curve analysis (**C,D**) of the prognostic value between the testing model and OC patients’ OS status when compared with or combined to clinical factors.

As for the testing cohort, we divided the group into 78 high-risk and 108 low-risk individuals based on the training cohort cut-off risk score. The outcomes of low- and high-risk groups’ patients of the testing cohort were also measured and the OS time of the high-risk group was significantly shorter than the lower-risk group (*P*=1.721e-02) (Figure 12E). The AUC of the prognostic model was 0.641 (Figure 12D). The risk scores distribution (Figure 12A) and survival status (Figure 12B) of OC patients, as well as the eight-prognostic gene expression heatmap (Figure 12C) in the testing group were also present. Meanwhile, the independency of the prognostic model was confirmed in testing cohort since univariate and multivariate Cox regression analyses showed the model correctly predicted high- or low-risk factor groups patients’ outcomes without relying on any clinical factors (Figure 13A,B). ROC curve analysis showed that the prognostic model exhibited better sensitivity and specificity when compared with tumor stage, grade and patients’ age for the AUC value of the model was much higher than later (Figure 13C). In accordance with results from training cohort, the combination of risk score and clinical factors showed better OS prediction capability (Figure 13D).

### The prognostic signature correlating to immune cells infiltration

**Figure 14:**
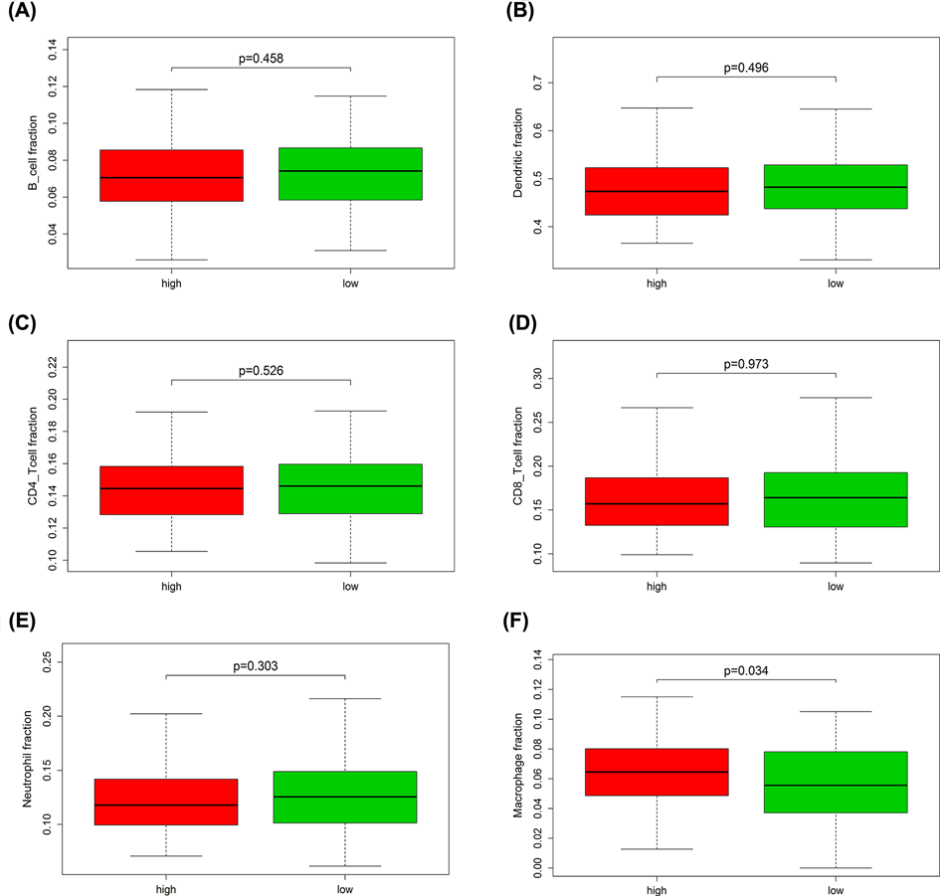
The expression level of immune cells related genes in high- and low-risk groups of the training cohort (**A**) B-cell fraction; (**B**) dendritic fraction; (**C**) CD4 T-cell fraction; (**D**) CD8 T-cell fraction; (**E**) neutrophil fraction; (**F**) macrophage fraction.

**Figure 15:**
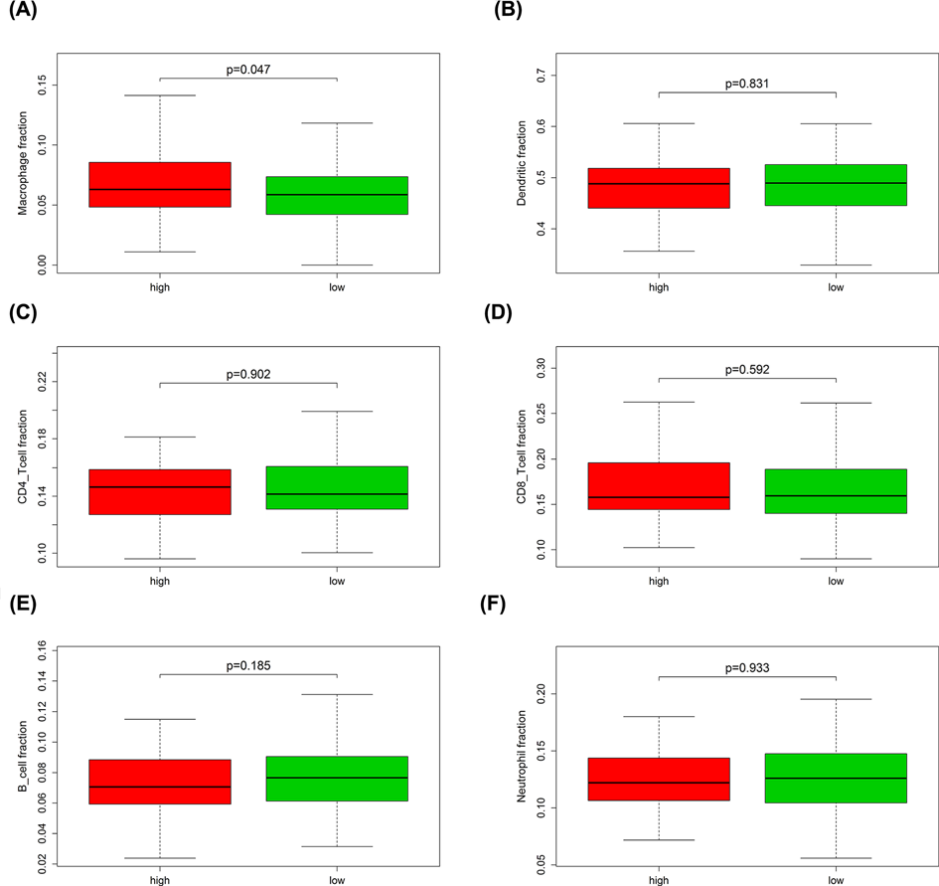
The expression level of immune cells related genes in high- and low-risk groups of the testing cohort (**A**) Macrophage fraction; (**B**) dendritic fraction; (**C**) CD4 T-cell fraction; (**D**) CD8 T-cell fraction; (**E**) B-cell fraction; (**F**) neutrophil fraction.

Through TIMER webtool, we analyzed the relative gene expression levels of six types of immune cells for each patient and found that genes concerning macrophage fraction were expressing significantly higher in the high-risk group (*P*<0.05) compared with the low-risk group in training cohort (Figure 14). Interestingly, same result was also observed in the testing cohort (Figure 15).

## Discussion

In the present study, we used the RRA methods to jointly analyze six GEO OC microarrays which contained 201 OC and 64 normal samples, identifying 605 DEGs and overlapped them with dysregulated genes of OC cohort from TCGA and GTEx portal, finally getting 164 up-regulated and 80 down-regulated genes. Functional analysis showed that 244 DEGs were significantly enriched in the cell division cycle, to be clear, in the process of the mitotic spindle. Spindle microtubules have been proved to play crucial role in physiological and pathological processes. As for cell division, only when all chromosomes linked to spindle microtubules through kinetochores and the spindle assembly checkpoint is satisfied, this process could step to anaphase [23]. Suraokar et al. found that the mitotic spindle assembly checkpoint and microtubule network were significantly altered in malignant pleural mesothelioma (MPM) while using epothilone-B, a non-taxane small molecule inhibitor targeting the microtubules, could greatly decrease the viability of MPM cell lines [24]. Rogalska et al. compared the anti-proliferative capacity of epothilone B with paclitaxel on OC cell line SKOV-3, found that this effect of Epo B was greater than latter [25]. The researches above were consistent with our study that the mitotic spindle process was dysregulated in OC progression, playing important roles in OC cell proliferation and tumor development.

PPI network construction of 244 DEGs included 238 nodes and 1284 edges, among which we identified three key modules. Interestingly, the top1 module was also highly associated with spindle microtubules and chromosome kinetochore, confirming the role of cell cycle in OC pathogenesis. The top ten hub genes from the PPI network were also recognized, which were *CDC45, CDK1, TOP2A, CDC20, CCNB1, CEP55, UBE2C, HMMR, FOXM1* and *TPX2*. Among them, CCNB1, UBE2C, CDK1, CEP55 as well as FOXM1 were found to be overexpressed in high-grade tumors and predicted worse outcomes. Besides, FOXM1, CDC20 and CCNB1 were the most frequently altered genes. These genes were reported to be closely associated with the BRCA1/2 mutation process of OC. It has been reported that females with BRCA1 or BRCA2 mutations were much more susceptible to get OC, accounting for the majority of the cohort [26]. Treszezamsky et al. found that BRCA1- and BRCA2-deficient cells are sensitive to Etoposide, which targeting topoisomerase II (TOP2A) and inducing DNA double-strand breaks [27]. High expression of CCNB1 was also observed in BRCA1-mutant cancer and induction of vinblastine targeting CCNB1 could significantly reduce tumor progression [28]. BRCA2 could interact with Filamin A actin-binding protein, further recruiting endosomal sorting complex required for transport (ESCRT)-associated proteins, Alix and Tsg101, and forming CEP55-Alix and CEP55-Tsg101 complexes at the midbody. The disruption of these processes by BRCA2 mutations results in increased cytokinetic defects, in part explain the instability of whole-chromosome in BRCA2-deficient OC and propose potential therapeutic target of CEP55 [29]. Olaparib, as a PARP inhibitor (PARP-i), has been widely used in BRCA1 or BRCA2 mutated OC patients’ treatment. However, Fang et al. found that Olaparib-induced adaptive response could be disrupted by FOXM1 while inhibiting FOXM1 by Thiostrepton could significantly enhance sensitivity to PARP-i. It is noteworthy that other genes were also been reported in previous studies of cancer. Yang et al. reported that CDC45 activated by DNA J heat shock protein family (Hsp40) member A1 (DNAJA1) could be reversed by KNK437 in colorectal cancer. The joint treatment of KNK437 with 5-FU/L-OHP chemotherapy significantly reduced liver metastasis of CRC. Cyclin-dependent kinase 1 (CDK1), a key regulator for cell cycle, was overexpressed in paclitaxel-resistant OC and predicted a poor OS [30], while miR-490-3P could reduce CDK1 expression, impeding OC cell proliferation [31] and Alsterpaullone could effectively reverse the drug-resistant trend [32]. Through searching CMap, we found Trichostatin A, pyrvinium and 8-azaguanine negatively correlated to the genomic-wide changes of OC. Trichostatin A has been proved to enhance the apoptotic potential of Palladium nanoparticles and increased the therapeutic potential in cervical cancer [33]. Likewise, co-treatment with BEZ235 and Trichostatin A enhanced autophagic cell death via up-regulating LC3B-II and Beclin-1 expression, finally exerting anti-tumor activity in breast cancer [34]. Pyrvinium was found to inhibit cell autophagy and promote cancer cell death. The combination of pyrvinium with autophagy stimuli improves its toxicity against cancer cells [35]. 8-azaguanine has also been used for treatment of various carcinoma, sarcoma, osteogenic sarcoma, lymphosarcoma and melanoma [36]. Hence, except for cisplatin or PARPi in treating OC, such small molecules may also reverse the malignant phenotypes of OC and serve as potential drugs for therapy.

By performing univariate and multivariate Cox regression analyses, as well as LASSO regression methods for 244 DEGs, we developed an eight-mRNA model that could classify OC patients into the high- and low-risk groups with significantly different OS. We explored the regulatory mechanism of eight-mRNAs in the signature by searching the published article, the majority of which were reported to be associated with tumorigenesis and tumor proliferation. DEFB1 is commonly considered as a single copy gene that encodes β-defensin 1 (BD-1), a member of the host defense peptide group. In human cancers, BD-1 is proposed to inhibit cell growth and promote apoptosis, acting as a tumor suppressor [37,38]. Zhang et al. found that forkhead box G1 (FOXG1) and miR-422a negatively regulated each other, forming a double-negative feedback loop to modulate the development and metastasis of hepatocellular carcinoma. KRTCAP3 was reported to be overexpressed in gastric cancer and human keratinocytes [39,40], while KLHL14 participated in the development and metastasis of endometrial cancer [41]. Besides, KLHL14 was also found to mutate in primary central nervous system lymphoma (PCNSL), playing a role in CNS development [42]. Kyle et al. proposed that SYNE4, as an outer nuclear membrane protein, could induce kinesin-mediated cell polarization [43]. The mutation of SYNE4 mediated the distinct disease phenotypes, acting as disease-causing behavior [44]. However, contrary to our observation, CXCR4 is observed to highly expressed in high-grade serous epithelial OC which positively related to tumor dissemination and metastasis while CCDC80 is down-regulated in papillary thyroid carcinomas and considered as a tumor suppressor role [45,46]. Treating with CXCR4 antagonists significantly inhibits tumor pro-invasive phenotype and knockdown of CCDC80 is susceptible to developed thyroid adenoma and OC [47,48]. Note that the role of TMC4 in OC pathogenesis has not been studied. This may offer a new direction for TMC4 in OC research. More recently, the prognostic value of mRNA-related signature has been reported in several studies [49,50]. However, current traditional clinical risk factors and clinical models have limited success in predicting OC patients’ outcomes due to the molecular heterogeneity and false-positive rate. Our LASSO regression model results with independent validation suggested that the combination of eight mRNA has good robustness and reproducibility in predicting prognosis for OC patients independent from traditional clinical risk factors, with the AUC marked 0.815, significantly higher than tumor stage, grade and patients’ age.

To investigate the biological function of various types of immune cells regarding OC, we explored the relative gene of each immune cell type and found that macrophage part expressed expressively higher in high-risk group in both training and testing cohort, pointing out the oncogenic-role of macrophages in OC development. Macrophage, as a type of immune-related cells, has already been considered to be closely associated with the malignant biological behavior of various cancers. M2 macrophage-like tumor-associated macrophages (TAMs) secreted EGF and then activated EGFR on tumor cells, further up-regulating VEGF/VEGF-R signaling in surrounding tumor cells to finally mediate OC cell proliferation and migration [51]. The exosomal miR-223 derived from macrophages under hypoxia condition reduced PTEN expression and led to increased PI3K/AKT signal activation, consequently mediated the drug resistance of EOC cells [52]. Hence, the potential therapeutic tools targeting macrophages may provide new perspective into OC treatment.

There are some highlights of our study. First of all, from the RRA integrated approach, we jointly explored six OC datasets in GEO databases and TCGA OC patients’ data matrix, finding some interesting niche factors and unique modules that were not seen earlier. Second, the present study discovered a multitude of DEGs and hub genes between OC and normal tissues, as well as the mutation condition of these genes. This information summarizes the genetic-level changes during the pathophysiological process of OC and provides possible target molecules for further research. Third, the prognostic model in our study can effectively predict OC patients’ outcomes, which provide a new method to help gynecologists evaluate patients’ prognosis in clinical practices.

However, some aspects of our study required improvement. First, our research was completely based on public data analysis, additional experimental studies are needed to explore the detailed molecular mechanism regarding DEGs and pathways, as well as the eight-mRNA prognostic model. Second, candidate drugs targeting hub genes and immune-related cells are needed to explore and clinical trials are also needed to verify whether the hub genes can be targeted to truly exert therapeutic effects and whether the prognostic model effectively predicts patients’ outcomes for OC. That said, with the ever-increasing accessibility and volume of genomic data from clinical patients and the continued development of technologies and algorithms, the bioinformatics analysis will further promote the progress of accurate diagnoses and personalized treatment in OC.

## Conclusion

In summary, in the present study, we identified 244 genes commonly dysregulated in OC, with 164 up-regulated and 80 down-regulated genes. The most enriched biological pathways regarding DEGs were cell cycle-related processes. From the PPI network concerning all DEGs which comprised 238 nodes and 1284 edges, we seek out top three hub modules and top ten hub modules. In addition, we found three molecular drugs may target OC for therapy. We also performed Cox and LASSO regression to present and validate a robust prognostic model aggregating eight-signature genes. Using this model, we could further distinguish patients with an elevated risk of mortality independent of other clinical factors, which may help us to improve our understanding of underlying mechanisms involved in OC and guide for diagnosing and prognosis prediction, as well as rational therapy in clinical practice.

## Supplementary Material

Supplementary Figures S1-S5

## Data Availability

The datasets used and/or analyzed during the current study are available from the corresponding author on reasonable request.

## Abbreviations

AUC: area under the ROC curve
BP: biological process
CC: cellular component
CMap: Connectivity Map
DEG: differentially expressed gene
EGF: Epidermal Growth Factor
EGFR: epidermal growth factor receptor
EOC: epithelial ovarian cancer
FDR: False discovery rate
FPKM: Fragments Per Kilobase of exon model per Million mapped fragments
GEO: Gene Expression Omnibus
GO: Gene Ontology
GTEx: Genotype-Tissue Expression
HPA: The Human Protein Atlas
KEGG: Kyoto Encyclopedia of Genes and Genomes
LASSO: least absolute shrinkage and selection operator
MCODE: molecular complex detection
MF: molecular function
MPM: malignant pleural mesothelioma
OC: ovarian cancer
OS: overall survival
PARP: poly ADP-ribose polymerase
PARP-i: PARP inhibitor
PPI: protein–protein interaction
ROC: receiver operating characteristic
RRA: robust rank aggregation
STRING: Search Tool for the Retrieval of Interacting Genes Database
TCGA: The Cancer Genome Atlas
TIMER: Tumor IMmune Estimation Resource
VEGF: vascular endothelial growth factor
VEGFR: Vascular Endothelial Growth Factor Receptor
