# Supplementary material for: Construction of a novel prognostic-predicting model correlated to ovarian cancer
Source: Biosci Rep. 2020 Aug 7;40(8):BSR20201261. doi: 10.1042/BSR20201261 (PMC7414523; doi:10.1042/BSR20201261)
Supplement: Supplementary Figures S1-S5 [file BSR-2020-1261_supp.pdf]

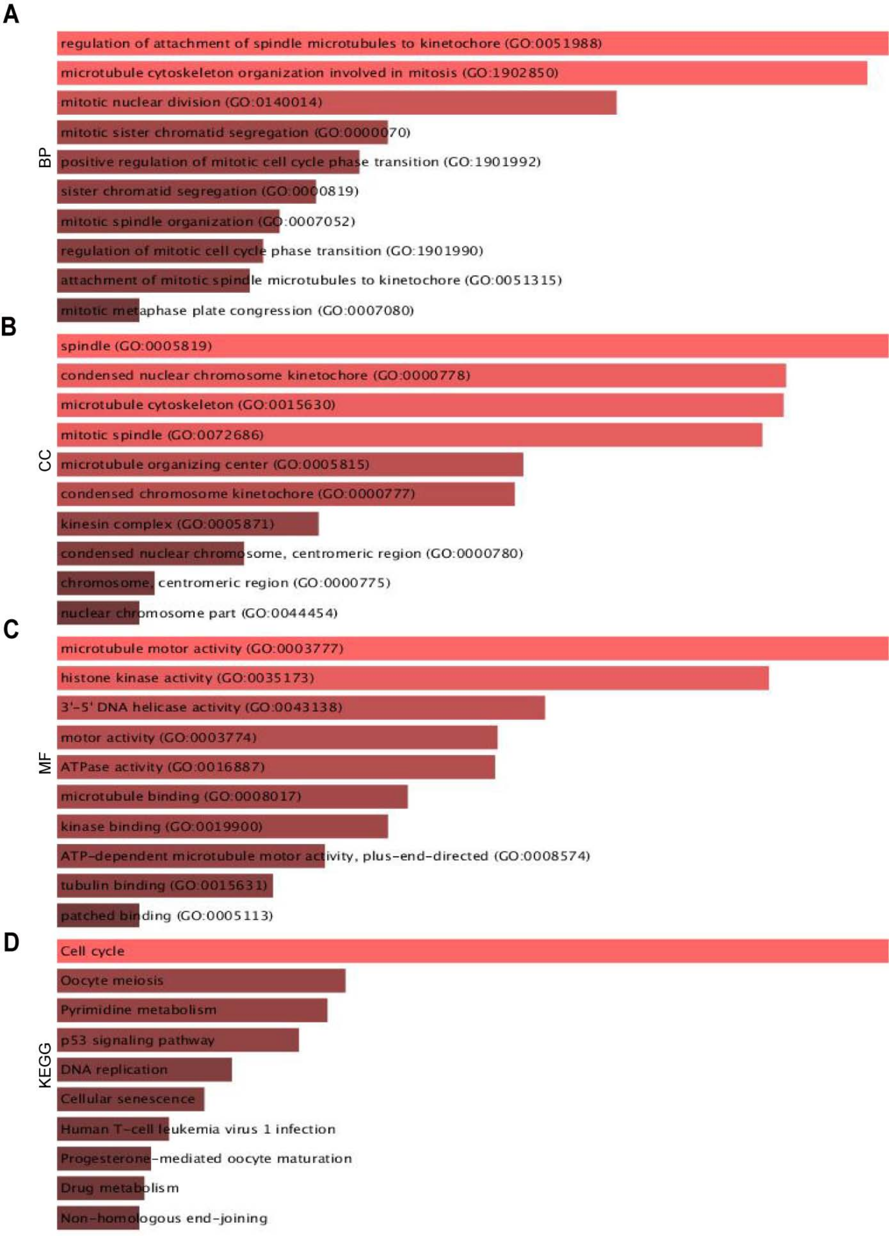
A
BP
regulation of attachment of spindle microtubules to kinetochore (GO:0051988)
microtubule cytoskeleton organization involved in mitosis (GO:1902850)
mitotic nuclear division (GO:0140014)
mitotic sister chromatid segregation (GO:0000070)
positive regulation of mitotic cell cycle phase transition (GO:1901992)
sister chromatid segregation (GO:0000819)
mitotic spindle organization (GO:0007052)
regulation of mitotic cell cycle phase transition (GO:1901990)
attachment of mitotic spindle microtubules to kinetochore (GO:0051315)
mitotic metaphase plate congression (GO:0007080)
B
CC
spindle (GO:0005819)
condensed nuclear chromosome kinetochore (GO:0000778)
microtubule cytoskeleton (GO:0015630)
mitotic spindle (GO:0072686)
microtubule organizing center (GO:0005815)
condensed chromosome kinetochore (GO:0000777)
kinesin complex (GO:0005871)
condensed nuclear chromosome, centromeric region (GO:0000780)
chromosome, centromeric region (GO:0000775)
nuclear chromosome part (GO:0044454)
C
MF
microtubule motor activity (GO:0003777)
histone kinase activity (GO:0035173)
3'-5' DNA helicase activity (GO:0043138)
motor activity (GO:0003774)
ATPase activity (GO:0016887)
microtubule binding (GO:0008017)
kinase binding (GO:0019900)
ATP-dependent microtubule motor activity, plus-end-directed (GO:0008574)
tubulin binding (GO:0015631)
patched binding (GO:0005113)
D
KEGG
Cell cycle
Oocyte meiosis
Pyrimidine metabolism
p53 signaling pathway
DNA replication
Cellular senescence
Human T-cell leukemia virus 1 infection
Progesterone-mediated oocyte maturation
Drug metabolism
Non-homologous end-joining

Figure S1. GO and KEGG functional annotation for module 1. (A) The top ten enriched biological process (BP) of the DEGs. (B) The top ten enriched molecular function (CC) of the DEGs. (C) The top ten enriched cellular component (MF) of the DEGs. (D) The top ten enriched KEGG pathways of the DEGs.

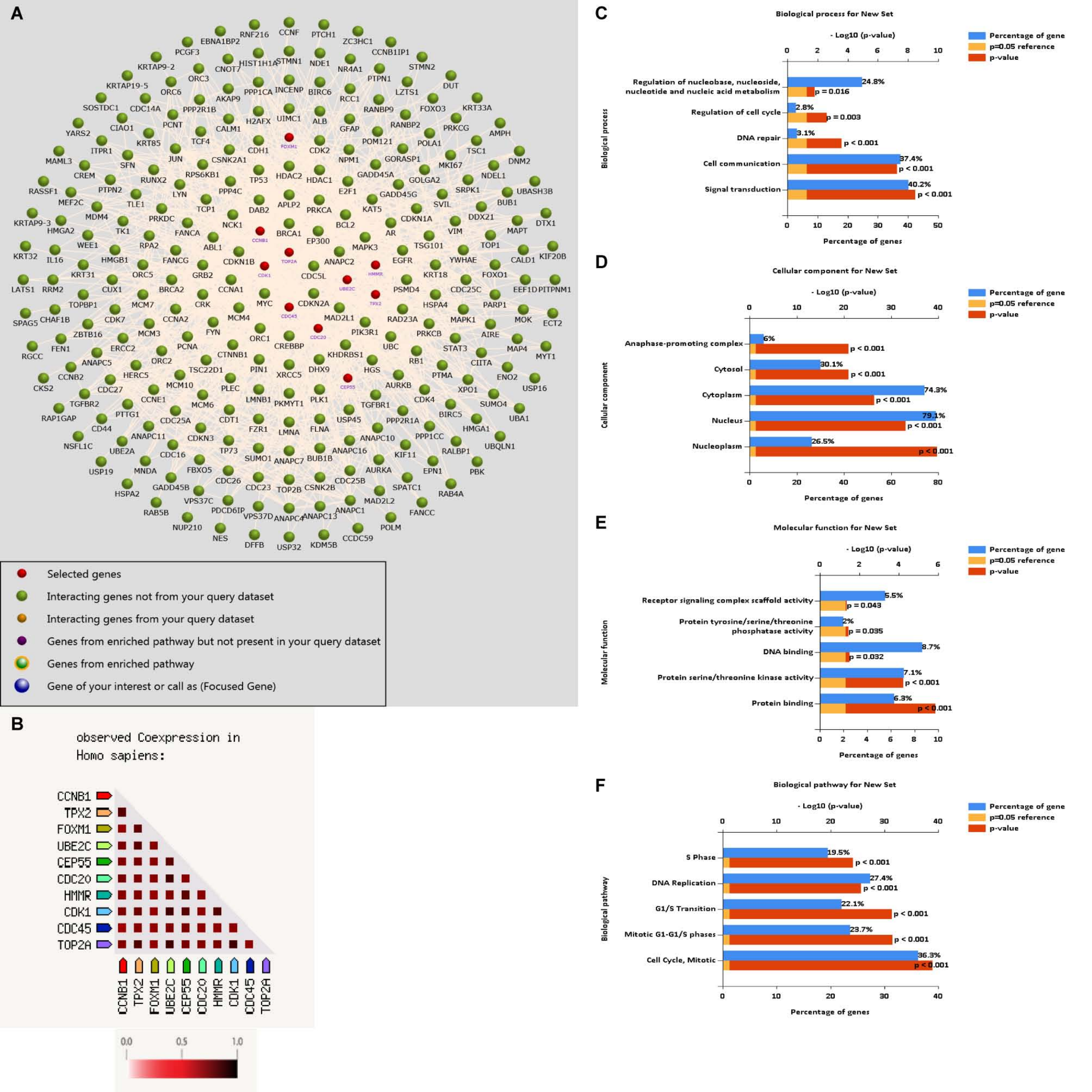
A
B
C
D
E
F
FOXM1
CCNB1
TOP2A
CDK1
HMMR
UBE2C
TPX2
CDC45
CDC20
CEP55
Selected genes
Interacting genes not from your query dataset
Interacting genes from your query dataset
Genes from enriched pathway but not present in your query dataset
Genes from enriched pathway
Gene of your interest or call as (Focused Gene)
observed Coexpression in
Homo sapiens:
CCNB1
TPX2
FOXM1
UBE2C
CEP55
CDC20
HMMR
CDK1
CDC45
TOP2A
0.0
0.5
1.0
Biological process for New Set
- Log10 (p-value)
Percentage of gene
p=0.05 reference
p-value
Biological process
Regulation of nucleobase, nucleoside, nucleotide and nucleic acid metabolism
24.8%
p = 0.016
Regulation of cell cycle
2.8%
p = 0.003
DNA repair
3.1%
p < 0.001
Cell communication
37.4%
p < 0.001
Signal transduction
40.2%
p < 0.001
Percentage of genes
Cellular component for New Set
Cellular component
Anaphase-promoting complex
6%
p < 0.001
Cytosol
30.1%
p < 0.001
Cytoplasm
74.3%
p < 0.001
Nucleus
79.1%
p < 0.001
Nucleoplasm
26.5%
p < 0.001
Molecular function for New Set
Molecular function
Receptor signaling complex scaffold activity
5.5%
p = 0.043
Protein tyrosine/serine/threonine phosphatase activity
2%
p = 0.035
DNA binding
8.7%
p = 0.032
Protein serine/threonine kinase activity
7.1%
p < 0.001
Protein binding
6.3%
p < 0.001
Biological pathway for New Set
Biological pathway
S Phase
19.5%
p < 0.001
DNA Replication
27.4%
p < 0.001
G1/S Transition
22.1%
p < 0.001
Mitotic G1-G1/S phases
23.7%
p < 0.001
Cell Cycle, Mitotic
36.3%
p < 0.001

Figure S2. The interaction network of ten hub genes with their related genes and its biological function. (A) The interaction network of ten hub genes; (B) The co-expression analysis of 10 hub genes; (C-E) Top five enriched biological process (BP), molecular function (CC) and cellular component (MF) of the interaction network. (F) Top five enriched KEGG pathways of the interaction network.

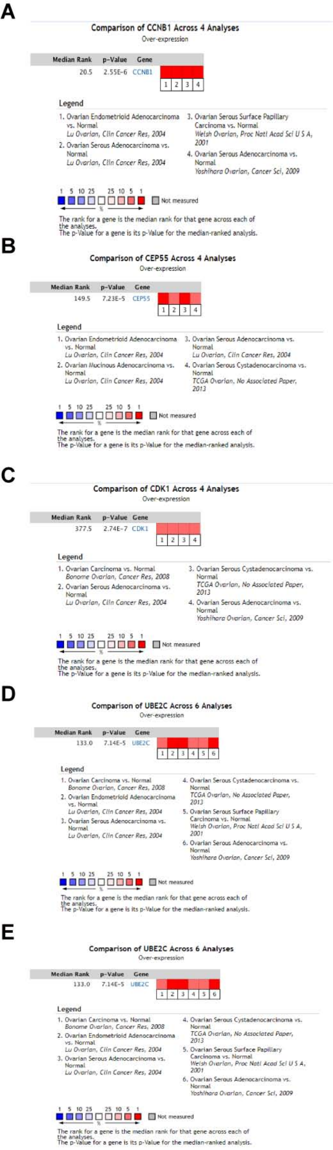
A
Comparison of CCNB1 Across 4 Analyses
Over-expression
Median Rank p-Value Gene
20.5 2.55E-6 CCNB1
1 2 3 4
Legend
1. Ovarian Endometrioid Adenocarcinoma vs. Normal
Lu Ovarian, Clin Cancer Res, 2004
2. Ovarian Serous Adenocarcinoma vs. Normal
Lu Ovarian, Clin Cancer Res, 2004
3. Ovarian Serous Surface Papillary Carcinoma vs. Normal
Welsh Ovarian, Proc Natl Acad Sci U S A, 2001
4. Ovarian Serous Adenocarcinoma vs. Normal
Yoshihara Ovarian, Cancer Sci, 2009
1 5 10 25 25 10 5 1
%
Not measured
The rank for a gene is the median rank for that gene across each of the analyses.
The p-Value for a gene is its p-Value for the median-ranked analysis.
B
Comparison of CEP55 Across 4 Analyses
Over-expression
Median Rank p-Value Gene
149.5 7.23E-5 CEP55
1 2 3 4
Legend
1. Ovarian Endometrioid Adenocarcinoma vs. Normal
Lu Ovarian, Clin Cancer Res, 2004
2. Ovarian Mucinous Adenocarcinoma vs. Normal
Lu Ovarian, Clin Cancer Res, 2004
3. Ovarian Serous Adenocarcinoma vs. Normal
Lu Ovarian, Clin Cancer Res, 2004
4. Ovarian Serous Cystadenocarcinoma vs. Normal
TCGA Ovarian, No Associated Paper, 2013
1 5 10 25 25 10 5 1
%
Not measured
The rank for a gene is the median rank for that gene across each of the analyses.
The p-Value for a gene is its p-Value for the median-ranked analysis.
C
Comparison of CDK1 Across 4 Analyses
Over-expression
Median Rank p-Value Gene
377.5 2.74E-7 CDK1
1 2 3 4
Legend
1. Ovarian Carcinoma vs. Normal
Bonome Ovarian, Cancer Res, 2008
2. Ovarian Serous Adenocarcinoma vs. Normal
Lu Ovarian, Clin Cancer Res, 2004
3. Ovarian Serous Cystadenocarcinoma vs. Normal
TCGA Ovarian, No Associated Paper, 2013
4. Ovarian Serous Adenocarcinoma vs. Normal
Yoshihara Ovarian, Cancer Sci, 2009
1 5 10 25 25 10 5 1
%
Not measured
The rank for a gene is the median rank for that gene across each of the analyses.
The p-Value for a gene is its p-Value for the median-ranked analysis.
D
Comparison of UBE2C Across 6 Analyses
Over-expression
Median Rank p-Value Gene
133.0 7.14E-5 UBE2C
1 2 3 4 5 6
Legend
1. Ovarian Carcinoma vs. Normal
Bonome Ovarian, Cancer Res, 2008
2. Ovarian Endometrioid Adenocarcinoma vs. Normal
Lu Ovarian, Clin Cancer Res, 2004
3. Ovarian Serous Adenocarcinoma vs. Normal
Lu Ovarian, Clin Cancer Res, 2004
4. Ovarian Serous Cystadenocarcinoma vs. Normal
TCGA Ovarian, No Associated Paper, 2013
5. Ovarian Serous Surface Papillary Carcinoma vs. Normal
Welsh Ovarian, Proc Natl Acad Sci U S A, 2001
6. Ovarian Serous Adenocarcinoma vs. Normal
Yoshihara Ovarian, Cancer Sci, 2009
1 5 10 25 25 10 5 1
%
Not measured
The rank for a gene is the median rank for that gene across each of the analyses.
The p-Value for a gene is its p-Value for the median-ranked analysis.
E
Comparison of UBE2C Across 6 Analyses
Over-expression
Median Rank p-Value Gene
133.0 7.14E-5 UBE2C
1 2 3 4 5 6
Legend
1. Ovarian Carcinoma vs. Normal
Bonome Ovarian, Cancer Res, 2008
2. Ovarian Endometrioid Adenocarcinoma vs. Normal
Lu Ovarian, Clin Cancer Res, 2004
3. Ovarian Serous Adenocarcinoma vs. Normal
Lu Ovarian, Clin Cancer Res, 2004
4. Ovarian Serous Cystadenocarcinoma vs. Normal
TCGA Ovarian, No Associated Paper, 2013
5. Ovarian Serous Surface Papillary Carcinoma vs. Normal
Welsh Ovarian, Proc Natl Acad Sci U S A, 2001
6. Ovarian Serous Adenocarcinoma vs. Normal
Yoshihara Ovarian, Cancer Sci, 2009
1 5 10 25 25 10 5 1
%
Not measured
The rank for a gene is the median rank for that gene across each of the analyses.
The p-Value for a gene is its p-Value for the median-ranked analysis.

Figure S3. The expression condition of 5 hub OS-related genes reported from various researches in Oncomine database. (A) CCNB1; (B) CEP55; (C) CDK1; (D) FOXM1;(E) UBE2C.

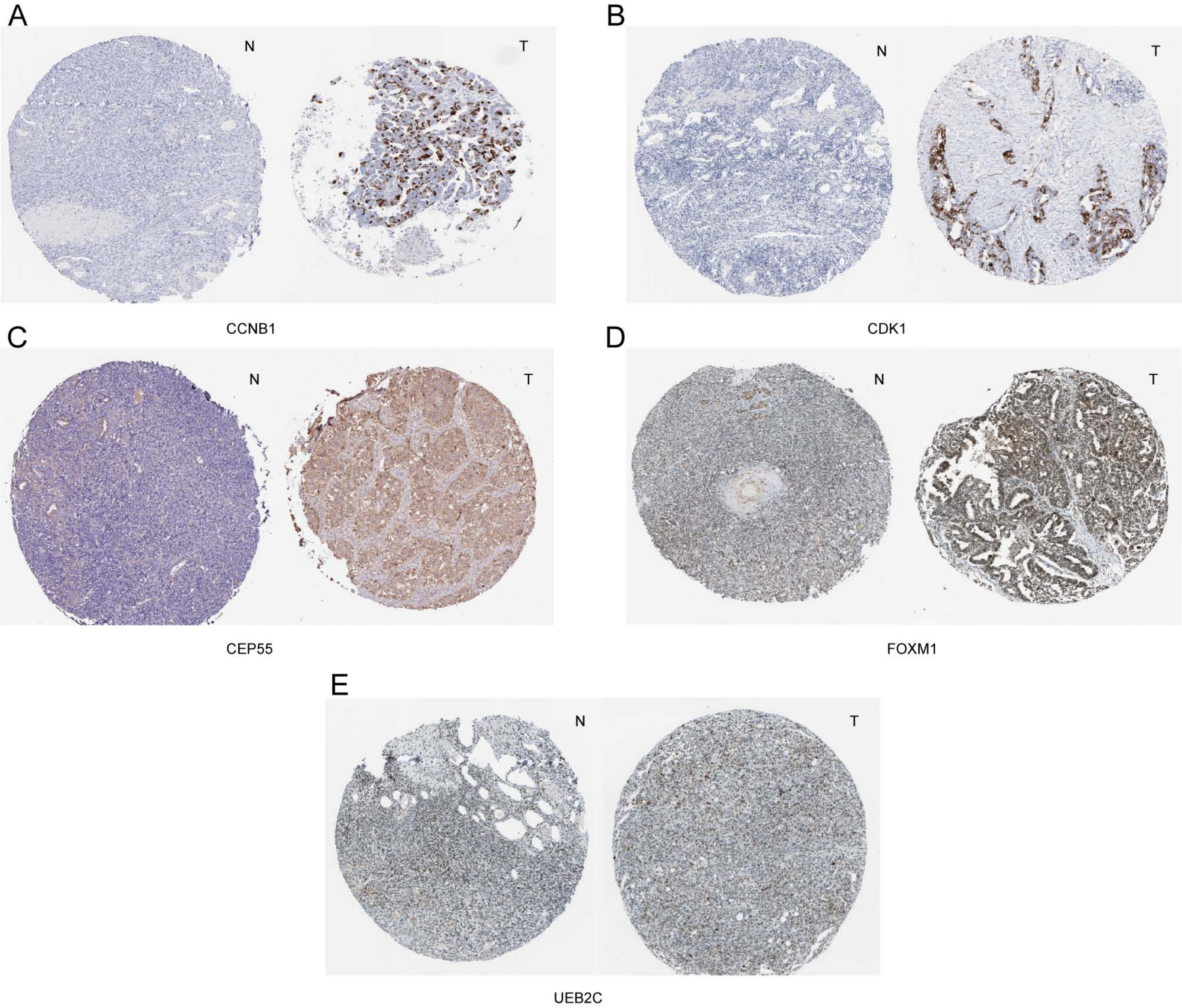
A
N
T
CCNB1
B
N
T
CDK1
C
N
T
CEP55
D
N
T
FOXM1
E
N
T
UEB2C

Figure S4. The immunohistochemistry of proteins translated from 5 hub OS-related genes between cancerous and normal ovary tissues in HPA database. (A) CCNB1; (B) CDK1; (C) CEP55; (D) FOXM1;(E) UBE2C. Abbreviation: N, normal; T, tumor; HPA, The Human Protein Atlas（HPA）database.

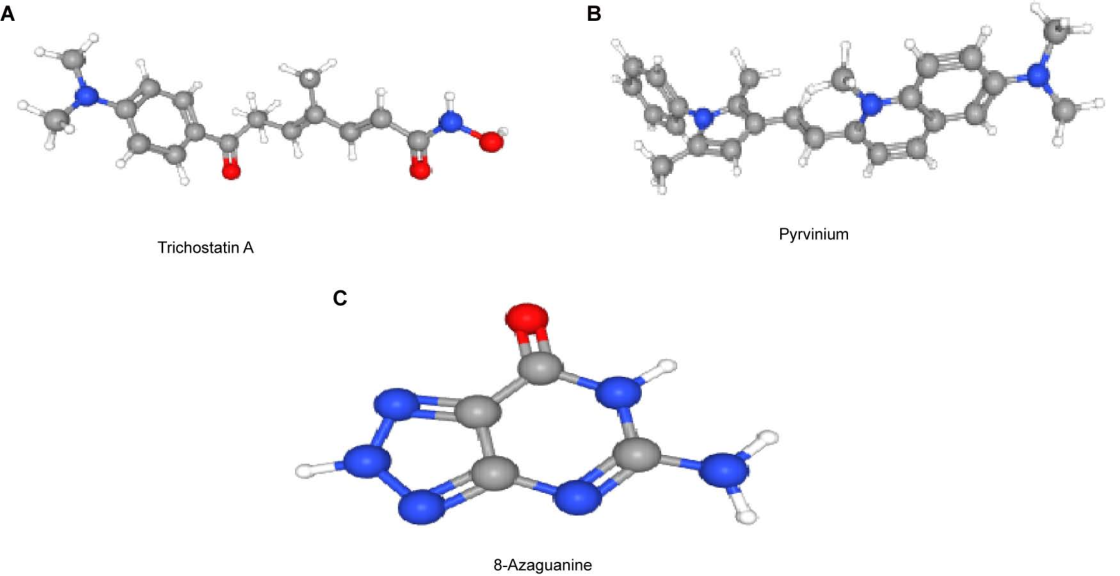
A
Trichostatin A
B
Pyrvinium
C
8-Azaguanine

Figure S5. The structures of three candidate molecular drugs from the Pubchem database (A) Trichostatin A; (B) pyrvinium; (C) 8-azaguanine.
